# Intemperance

**Published:** 1875-01

**Authors:** 


INTEMPERANCE.—An official statement has been made that within three years, sixteen times more women than men have been imprisoned for drunkenness in the City of New York. It is well known that the entire earnings of thousands of laboring men are spent for liquor and tobacco, which are not only useless, but actually hurtful, and, in many cases, ruinous. Six hundred millions of dollars are spent in the United States annually for liquor in its various forms. Suppose for one year not a dollar was spent in this direction, how much more would be left for the working people of the country to purchase better clothing, better food, better boots, and better houses for their families? The business community would be that much richer every year; hence, every merchant, every trader, every man who buys and sells, is pecuniarily interested in passing laws, and by their influence and example in having them executed for the curtailment of the liquor traffic. Besides all this, men who do not drink do more work, and do it better, than those who do? A manufacturing firm in New England found that, in one year, three hundred and seventy-five men who used no liquor, earned eight per cent. more than four hundred men who did use it, making altogether a difference in yield of fifteen per cent. Until the millennium, principles will never restrain the masses; nor will principles restrain one quarter of our population. Temperance societies have done great good, especially by leading to the enactment of laws for the curtailment of the manufacture and trade in whatever can intoxicate. Perhaps the time may come when the power to make and sell liquors shall be reserved to the State alone.

---

Dirt is disease; cleanliness is health.

THE WHIP AND SPUR.—That man or woman who, when feeling too weak to perform the allotted task, flies to some brain stimulant to secure the temporary strength, is certainly a very thoughtless person, if not almost a suicide. It is this practice of seeking power from wine or brandy, or whiskey or tobacco, or coffee, or tea, or opium, which brings sudden death to so many gifted men. It is this habit—the habit of working on stimulants—which makes a multitude of drunkards. The best possible thing for a man to do when he feels too tired to perform a task, or too weak to carry it through, is to go to bed and sleep a week if he can; this is the only true recuperation of brain-power; the only actual renewal of brain forces, because during sleep the brain is, in a sense, at rest, in a condition to receive and appropriate particles of nutriment from the blood which take the place of those which have been consumed in previous labor. Mere stimulants supply nothing; they only goad the brain, force it to a greater consumption of its substance, until that substance has been so fully exhausted that there is not power enough left to receive a supply; just as men are sometimes so near death by thirst and starvation that there is not strength enough left to swallow anything, and all is over. The true way is to rest the brain when it is exhausted; and renewed force comes to it in no way so surely and so rapidly as in early and abundant sleep. To so inflame the brain by overwork as to deprive it of the power of repose and recuperation, is almost certain death, and is absolutely certain and permanent injury to body and brain.

---

The worst of all the bad forms in which alcohol presents itself is that of ale or beer. The true name for it, though not a pleasant one, is *swill.*

